# A modified QuickDASH-9 provides a valid outcome instrument for upper limb function

**DOI:** 10.1186/1471-2474-10-161

**Published:** 2009-12-18

**Authors:** C Philip Gabel, Michael Yelland, Markus Melloh, Brendan Burkett

**Affiliations:** 1 Faculty of Science, Centre for Healthy Activities, Sport and Exercise, University of the Sunshine Coast, Queensland, Australia; 2 Primary Health Care Section, School of Medicine, Griffith University, Queensland, Australia; 3 Section of Orthopaedic Surgery, Department of Medical and Surgical Sciences, Dunedin School of Medicine, University of Otago, Dunedin, New Zealand

## Abstract

**Background:**

The 30-item Disabilities Arm Shoulder and Hand (DASH) questionnaire was introduced to facilitate assessment of upper limb functional limitations. To improve practicality and eliminate item redundancy a modified instrument was needed. The 11-item QuickDASH was developed to fulfil these requirements and translated into several languages. However, prospective investigations of psychometric and practical characteristics are limited. No published study investigated readability or used concurrent validation with a standardized upper limb criterion measure. The validity of the QuickDASH has been questioned as the results for factor structure are conflicting, and the English-language version has not yet had factor structure reported. A shortened 9-item version, the QuickDASH-9, that addresses these issues is proposed.

**Methods:**

This two-stage observational study assessed the psychometric and practical characteristics of the QuickDASH and the extracted QuickDASH-9. The Upper Limb Functional Index (ULFI) was the criterion standard in both stages. Stage 1, calibration, reanalyzed extracted QuickDASH and QuickDASH-9 responses from a previous prospective study, by the authors, of the 30-item DASH (n = 137). Stage 2, prospective validation, investigated the QuickDASH through repeated measures in consecutive upper limb musculoskeletal participants' consulting for physical therapy in Australia (n = 67). The QuickDASH and extracted QuickDASH-9 data from both stages was analyzed and compared for psychometric properties, practical characteristics and factor structure.

**Results:**

The proposed QuickDASH-9 had a unidimensional structure, high reliability (ICC 2:1, r = 0.92), internal consistency (alpha = 0.93) and responsiveness (ES = 1.05). It correlated highly with both the DASH (r = 0.97), QuickDASH (r = 0.99) and ULFI criterion (r = 0.85). QuickDASH-9 missing responses reduced to 3.5% from 26% in the QuickDASH. Completion and scoring time was 134 ± 56 seconds and required a computational aid. The QuickDASH demonstrated a bidimensional structure making it invalid. The QuickDASH-9 summary performance was measured on the 'Measurement of Outcome Measures' at 88% and on the 'Bot' clinimetric scale at 75%.

**Conclusions:**

The proposed QuickDASH-9 had a unidimensional structure and similar psychometric precision to the full-length DASH with improved practicality and completion time. The QuickDASH was invalid as its bidimensional structure made a single summated score inappropriate. The QuickDASH-9 offers a future direction for ongoing use of the QuickDASH concept.

## Background

The assessment process in both the clinical and research setting has progressively incorporated patient-reported outcome (PRO) measures and upper limb assessment is no exception. Regional and condition specific PROs enable the quantification of patient impairment [1]. Doward and McKenna have described this as a '...needs based approach' [2]. This assists the clinical decision-making process [3,4] and facilitates compliance with the protocols within professional organizations [5], government agencies [6,7] and insurer groups [8]. There are limited upper limb PROs developed specifically for the region as a single kinetic chain [9] that accommodate the requirements of both the clinician and researcher in an efficient and effective manner [10,11].

The 30-item Disabilities Arm Shoulder and Hand (DASH) [9] is reported to fulfil these criteria. It was validated for a variety of disorders [1,9,12-16] and its availability in different languages has increased rapidly [17-19]. The shorter 11-item QuickDASH was developed to reduce respondent and administrative burden and eliminate item redundancy. This improved compliance [20], item redundancy and scale width for higher impairment conditions [21]. Consequently, there is an impetus for the QuickDASH to replace the DASH [22] and be advocated as a criterion standard for upper limb measurement [23,24]. However, the validity of the QuickDASH has been questioned as a consequence of conflicting findings on the factor structure [25,26]. A single factor structure is an essential property of all PROs that provide a single summated score [27]. A PRO must exhibit a single predominant theme or factor, such as upper limb function, that is common to all item-questions. The factor structure must be unidimensional when analyzed. The most appropriate method is Maximum Likelihood extraction (MLE) [28].

A literature search (PubMed, Medline, CINAHL, Embase, Cochrane and Google Scholar) found five prospective studies that investigated the QuickDASH. They considered the psychometric and practical characteristics in general populations [24,25,29], burns patients [30] and as a work injury prediction tool [31]. The original validation [20] and several subsequent studies reanalyzed data with the eleven items extracted from existing 30-item DASH responses [21,26,32]. Only two studies investigated the QuickDASH factor structure. There was a unidimensional structure in the prospective study on the Japanese-language version [25] but a bidimensional structure in reanalyzed extracted data of the French-language version [26]. Both authors used principal component analysis which is considered inappropriate for PROs [28]. Factor structure in the English-language version has not been reported. Consequently, the factor structure must be clarified and determined prospectively with appropriate item-extraction methodology in a general upper limb population.

The primary aim of this study was to determine the factor structure of the QuickDASH and QuickDASH-9. If unidimensional and valid, the next step was to calibrate and validate the psychometric properties and practical characteristics in independent general upper limb populations. Finally these characteristics were compared and correlated with the original full-length DASH and a validated criterion standard, the Upper Limb Functional Index (ULFI) [11,33].

## Methods

### Development of the QuickDASH-9

**Figure 1:**
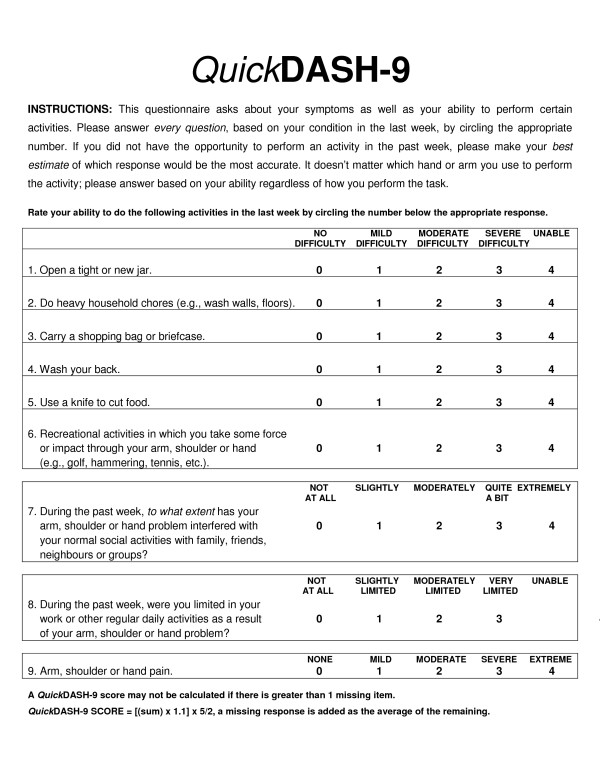
**QuickDASH-9**.

The concept-retention methodology used to reduce the full-length DASH to the QuickDASH [20,34] was employed to produce the QuickDASH-9 (Figure 1). The authors used consensus agreement following feedback from a practicality focus-group composed of 20 patients and five therapists. This ensured face and content validity would be consistent with the QuickDASH. Items #10 (Pins and needles) and #11 (Sleep) were removed as neither are an activity of daily living. It was hypothesised these changes would enable the QuickDASH-9 to exhibit a unidimensional factor structure. The scoring system was also modified from the existing 1-5 scale to a 0-4 scale and the calculation for scoring adjusted accordingly.

### Design

**Figure 2:**
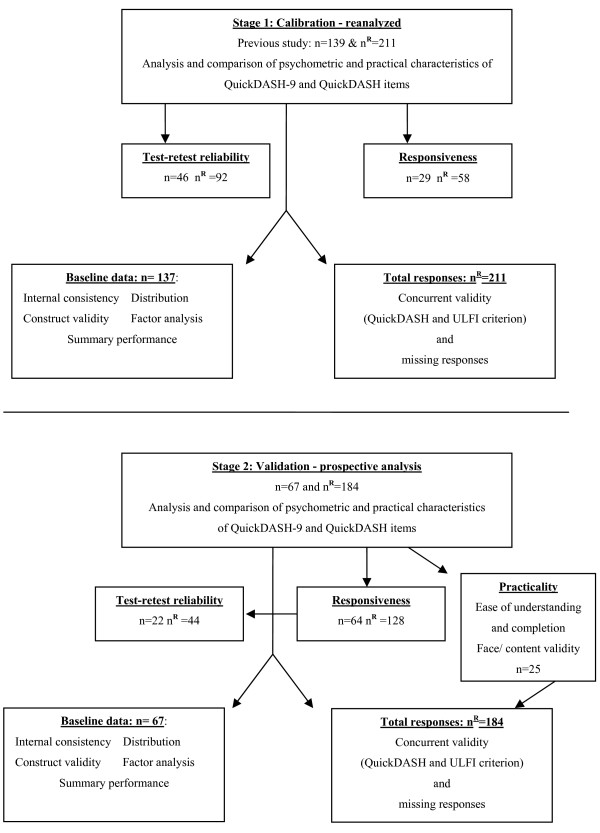
**Flow chart of calibration from stage 1 and validation stage 2**. All QuickDASH-9 data was extracted from the QuickDASH; n = total number of participants; n^R^ = total number of responses; practicality n = 25 composed 20 patients and 5 therapists.

A two stage observational study was used. Stage 1, calibration, extracted the items from the DASH responses in a previous study [11] to form the QuickDASH-9 and QuickDASH. Stage 2, prospective validation, concurrently measured the QuickDASH and ULFI. The QuickDASH-9 scores were determined from extracted QuickDASH responses (Figure 2).

### Assessment Questionnaires

The DASH is a four-page 30-item PRO on a 5-point Likert scale (1-5). Subsequent raw scores range from 30 to 150 and are converted to a percentage, 0 (no disability) to 100 (most severe disability) [9]. It has two optional sport or music and work scales, not used in this study. Up to three missing responses are permitted [35]. The QuickDASH is a single-page PRO with eleven items extracted from the DASH [20]. It uses the DASH scale and scoring method and allows for one missing response [9].

The QuickDASH-9 is a single-page PRO with nine items extracted from the QuickDASH and DASH. It uses the DASH scoring method on a 0-4 Likert scale and allows for one missing response (Figure 1).

The ULFI is a single-page 25-item PRO on a 3-point Likert scale. Subsequent raw scores range from 0-25 and are multiplied by four to provide a percentage scale, 0 (normal) to 100 (maximum impairment). Up to two missing responses are permitted [11,33]. It has an 11-point global 'Numeric Rating Scale' (NRS) to assess overall status with anchors of 0 ('normal or pre-injury') to 10 ('worst possible'). Two optional components provide a qualitative 'Patient Specific Index' and a self-assessed ranking of duties that can be used to calculate a 'Global Assessment of Body And Limbs' score [36].

### Setting and Participants

Participants with upper limb musculoskeletal conditions under referral from a medical practitioner were recruited consecutively or successively from primary care physical therapy outpatient clinics. These conditions included soft tissue injury, post surgery, lymphoedema, fractures, chronic regional pain and trauma. Exclusion criteria were <18 years of age, difficulty with English language comprehension and cognitive impairment. Symptom duration ranged from one week to eight years with a mean of 38.7 ± 41.6 weeks. Removal of one outlier at eight years reduced the mean duration to 20.3 ± 25.2 weeks.

Participants receiving ongoing treatment during both the calibration and validation stages were measured at baseline, then at two weekly intervals for six weeks, then four weekly thereafter until discharge. Status was classified as: acute - injured within the previous six weeks; subacute - six to twelve weeks; and chronic - greater than twelve weeks [37].

### Study Stages

#### Stage 1, calibration

Existing data was reanalyzed. This included 211 responses from 137 participants from nine physical therapy outpatient centres in three different Australian states. The methodology is described in a previous publication by the authors [11]. Demographic details are presented in Table 1.

**Table 1:** Participant demographics

Demographics	Stage 1 Calibration (Gabel et al 2006)	Stage 2 Validation ULFI, QuickDASH
**Participants **(n)	137	67
**Responses **(n)	211	184
**Age **(years)	48.4 ± 15.6	48.3 ± 18.6
**Gender: **Female (%)	54	35
**Dominance: **Right (%)	77	93
**Injury: **Duration (weeks)	24.5 ± 28.8	11.7 ± 17.8
Time range (weeks)	1 - 433	1 - 80
**Work status: **Employed (%)	61	57
Retired (%)	0	36
Unemployed (%)	39	7
**Injured at work **(%)	40	23
**On work cover **(%)	30	23

#### Stage 2, validation

A prospective investigation examined 184 responses from 67 participants, recruited from six physical therapy outpatient centres in one Australian state. Demographic details are presented in Table 1. Repeated measures were made for subgroups of responsiveness (n = 64) and reliability (n = 22). This provided prospective investigation of the concurrently completed QuickDASH and ULFI to determine psychometric and practical characteristics (Figure 2). All QuickDASH-9 responses were extracted from the QuickDASH.

### Analysis - Methodological Characteristics

#### Test-retest reliability

The ICC (2:1) [38] was used at 72 hours from baseline during a period of non-treatment with the NRS as an external reference [3,9,11].

#### Responsiveness

Effect size (ES) and standard response mean (SRM) were used [39]. The NRS provided an external reference standard. Two compared measures were taken. The first at baseline, with the repeated measures made following a period of anticipated change due to natural healing and therapist intervention. These periods were consequently a partial duration of the injury classification being: two weeks for acute participants, four weeks for subacute and six weeks for chronic [9,11,40].

#### Measurement error

The minimal detectable change was taken at the 90% level (MDC_90_) [41].

#### Validity

Face and content validity were determined from the development studies [11,20,21] and supported in this study by the practicality focus-group (Figure 2). Criterion or concurrent validity was assessed using a Pearson correlation coefficient. Construct validity was demonstrated by a standard t-test that verified change between the baseline and the repeated measures [11,20].

#### Internal consistency

Cronbach's alpha coefficient was used [42,43].

#### Distribution and normality

This was determined through inspection of the histograms and the one-sample Kolmogorov-Smirnov (KS) test [44].

#### Factor analysis

The MLE method was used [28] with varimax rotation if two or more factors were determined and coefficient suppression was set at 0.5 [44,45]. Factor extraction was determined *a-priori *by: the scree-plot curve point of inflection [46]; an eigenvalue cut-off of 1.0 [47]; and that ≥ 10% of total explained variance was accounted for where average communality after extraction was ≥ 0.6 [45].

#### Sample size

To ensure sufficient sample power to provide an 80% confidence level in determining actual change above 10.5%, the MDC_90 _for the DASH, the Dawson and Trapp method was used [48] where required, sample size (n) is:

(U1-U0) = clinically important difference between the means; SD = standard deviation in the population. Za = two tailed and Zb = lower tail as defined from Tables of significance levels.

### Analysis - Practical Characteristics

#### Missing responses

These were noted as a percentage of total responses.

#### Completion and scoring time

These were calculated from the average of three separate tests in the practicality focus group.

#### Readability

The Flesch-Kincaid reading scale was used to determine ease of comprehension and readability [49,50] and calculated from the grammar function from within the word processing program.

#### Summary performance

Two clinimetric scales were used. The 25-item 'Measurement of Outcome Measures' that considered a measures characteristics under four categories: methodological, practical, distributional and general. The total was summated and multiplied by four to provide scores from 0 to 100% [11]. The 12-item 'Bot scale' considered twelve individual practical and methodological characteristics of a measure and is scored on a 0-12 scale that can be converted to a percentage [16].

#### Statistical analysis

The Statistical Package for Social Sciences version 14.0 (SPSS Inc, Chicago, IL) was used to analyze the data on an intention-to-treat principle. Statistical sigificance was accepted at the *p *< 0.05 level. Pooled samples of each questionnaire enabled determination of distribution, missing responses, internal consistency and factor analysis.

#### Ethics

Ethics approval was given by the University of the Sunshine Coast Human Research Ethics Committee.

## Results

### Factor Structure

The QuickDASH-9, DASH and the ULFI each had a unidimensional structure determined for their factor matrix in both stages, so no varimax rotation occurred. The QuickDASH had a bidimensional structure, invalidating any single summated score and precluding any further valid analysis of its psychometric properties.

Factor loadings for all items in both the QuickDASH and QuickDASH-9 exceeded the 0.50 suppression level. In the calibration stage the QuickDASH-9 and QuickDASH had primary eigenvalues of 5.4 and 5.7 respectively which accounted for 54% and 62% of variances. In the validation stage these increased respectively to eigenvalues of 6.1 and 6.5 which accounted for 61% and 59% of variance. The QuickDASH-9 factor order was consistent apart from question-item #5 'Use knife' which loaded sixth in the calibration and first in the validation stage (Table 2). The QuickDASH demonstrated identical factor order in both stages (Table 3); however, in addition to the invalid bidimensional structure, one item 'Limited in work' changed factors and another 'Socialize' had cross-loading in the validation stage.

**Table 2:** QuickDASH-9 factor matrix

Stage 1 Calibration (n = 137)		Factor		Stage 2 Validation (n = 67)	
Question #	Item	1	1	Item	Question #
2	Heavy chores	.796	.850	Use knife	5
3	Carry bag	.744	.828	Heavy chores	2
8	Limited in work	.743	.790	Limited in work	8
1	Open jar	.704	.782	Open jar	1
6	Forceful recreation	.701	.769	Carry bag	3
5	Use knife	.696	.749	Wash back	4
7	Socialize	.667	.718	Forceful recreation	6
4	Wash back	.645	.709	Socialize	7
9	Pain intensity	.565	.648	Pain intensity	9

Maximum likelihood extraction with suppression at 0.50. No varimax rotation as only 1-factor is extracted.

**Table 3:** QuickDASH rotated factor matrix

Stage 1 Calibration (n = 137)		Stage 1 Factors		Stage 2 Factors		Stage 2 Validation (n = 67)	
Question #	Item	1	2	1	2	Item	Question #
2	Heavy chores	.823		.906		Heavy chores	2
1	Open jar	.709		.888		Open jar	1
5	Use knife	.661		.701		Use knife	5
3	Carry bag	.642		.684		Carry bag	3
6	Forceful recreation	.618		.621		Forceful recreation	6
4	Wash back	.616		.567		Wash back	4
8	Limited in Work	.589			.859	Limited in work	8
9	Pain intensity		.894		.808	Pain intensity	9
11	Sleep		.736		.775	Sleep	11
10	Pins and needles		.539		.680	Pins and needles	10
7	Socialize		.512	.543	.615	Socialize	7

Maximum likelihood extraction and varimax rotation with suppression at 0.50.

### Psychometric properties

These are presented for each PRO in Table 4 with the construct validity in Table 5. The values for the QuickDASH are invalid but are provided as a comparison to the other PROs and to the findings of previous QuickDASH studies.

**Table 4:** Methodological characteristics of QuickDASH-9, QuickDASH, DASH and ULFI

	Reliability	Internal consistency	Error score		Responsiveness			Missing responses
Stage	Rxx (ICC)	Alpha	SEM	MDC_90_	SD_100_	ES	SRM	Percentage
**Calibration**	(n = 46)	(n = 139)	(n = 29)	(n = 29)	(n = 29)	(n = 29)	(n = 29)	(n = 137)
**QuickDASH-9**	0.94	0.89	4.82	11.22%	20.05%	1.19	1.65	11%
**QuickDASH**	0.94	0.92	4.98	11.58%	20.71%	1.21	1.75	12.5%
**DASH**	0.98	0.96	2.84%	6.63%	19.67%	1.41	2.18	34%%
**ULFI**	0.96	0.89	4.50%	10.50%	21.61%	1.28	1.87	<0.5%
**Validation**	(n = 22)	(n = 67)	(n = 64)	(n = 64)	(n = 64)	(n = 64)	(n = 64)	(n = 67)
**QuickDASH-9**	0.94	0.925	7.38%	17.18%	26.07%	1.05	1.21	3.5%
**QuickDASH**	0.91	0.92	6.73%	15.66%	23.20%	1.05	1.23	26.5%
**ULFI**	0.98	0.92	3.41%	7.93%	24.16%	0.93	1.25	<0.5%

The QuickDASH psychometric properties are invalid. They are provided as a comparison to the other PROs and to the findings of previous QuickDASH studies.

SD100: Standard deviation at baseline (100% scale); Rxx: Test-retest reliability coefficient; ICC: Intra-class Correlation Coefficient for test-retest reliability; SEM: Standard Error of the Measurement; MDC90: Minimal Detectable Change (90% CI); ES: Effect Size; SRM: Standard Response Mean; Alpha: Cronbach's Alpha.

**Table 5:** Construct validity comparison between baseline and repeated measures

Stage	Baseline Mean	Repeat Test * Mean	Paired *t-*Stat **
**Calibration** **Sample n = 31**			
**QuickDASH-9**	54.9 ± 20.5	46.9 ± 26.6	5.2
**QuickDASH**	58.1 ± 20.8	40.6 ± 23.1	5.7
**DASH**	51.6 ± 20.5	34.7 ± 22.2	6.4
**ULFI**	58.1 ± 23.0	41.3 ± 26.6	5.6
**Validation** **Sample n = 64**			
**QuickDASH-9**	47.0 ± 26.1	23.2 ± 18.6	4.1
**QuickDASH**	44.4 ± 23.2	20.0 ± 16.1	4.2
**ULFI**	41.5 ± 24.6	19.8 ± 19.1	7.6

* Repeated measures made following a period of anticipated change due to natural healing and therapist intervention: at two weeks for acute participants, four weeks for subacute and six weeks for chronic [9,11,39].

** p value <0.001 for all *t*-statistic measures.

### Distribution

The impairment range of 0-100% was shown for all PROs with the number of 5% histogram increments for the total score being QuickDASH-9 = 19, QuickDASH = 18 and DASH = 17 whilst the ULFI had values in all 20 increments.

### Practical Characteristics

#### Missing responses

These are detailed in Table 4.

#### Completion and scoring times

The QuickDASH-9 and QuickDASH were respectively 134 ± 56 seconds and 155 ± 64 seconds and both required a computational aid. The ULFI was 132 ± 51 seconds.

#### Readability

This was found at grade twelve for the QuickDASH-9 and at grade seven for the ULFI.

#### Summary performance

The 'Measurement of Outcome Measures' score for the QuickDASH-9 was 88%, the DASH was 72% and the ULFI was 96%. On the 12-point Bot scale the score for the QuickDASH-9 was nine (75%), the DASH was seven (58%) and the ULFI was twelve (100%). The QuickDASH was invalid with respective clinimetric scores of 44% and three (25%).

## Discussion

This study proposes the QuickDASH-9, with its valid unidimensional structure, as a way to overcome the existing shortcomings of the QuickDASH. This will enable the concept to continue. The modifications that produce the QuickDASH-9 fulfil the original aims of the QuickDASH [20]: a shortened version of the full-length DASH with comparable or preferable psychometric properties, improved practicality and the elimination of item redundancy [9,11]. In attempting to achieve these aims the QuickDASH produced a bidimensional factor structure. Its validity as a single summated score cannot be supported.

Our findings propose the DASH scoring scale of 1-5 be modified to 0-4 in the QuickDASH-9. This uses the established format of a 0 based anchor rather than a 1 [51]. This should facilitate practicality and ensure consistency of scoring with other PROs.

The bidimensional structure of the QuickDASH, demonstrated in this study using MLE, is consistent with previous findings by Fayad [26] but conflicts with the unidimensional structure found by Imaeda [25]. However, both previous researchers used principal component analysis which is not recommended [28]. In this study the QuickDASH bidimensional structure demonstrated two factors that can be broadly divided into 'activity' and 'non-activity' items which supports previous findings [21,26]. The original DASH has a unidimensional structure [33,52-54]. This means the reductive process of concept-retention methodology, that reduces the DASH's 30 items to eleven in the QuickDASH, causes a fundamental change in the factor structure [55]. It is critical that a PRO exhibits a unidimensional structure if it is to accurately reflect the measured region with a single summated score [27].

There is a distinct lack of prospective studies of the QuickDASH and no English versions were found that investigated factor structure. Furthermore, reporting of psychometric properties is incomplete if the factor structure is not stated [24,29-31], and consequently misleading and the results invalid if the structure is not unidimensional.

The use of extracted items from the DASH as the sole method to validate the QuickDASH without prospective testing [20,21,26,32], should only be investigatory. This methodology risks shared measurement error and does not account for part or whole correlation [56] which can lead to type I errors [43]. By completing the prospective aspect of this study on a general upper limb population with a consistent regional reference standard, the ULFI, these error concerns are alleviated for the QuickDASH. However, for the QuickDASH-9 the same criticism applies as it is investigatory research only.

Should the findings of this study be supported by further research, then the QuickDASH-9 would be appropriate to replace the QuickDASH and also the original DASH. Similar proposals are already in place in other body regions. The Neck Disability Index, an advocated PRO, was recently shown to be invalid due to its bidimensional structure [57,58]. It is proposed that a shortened unidimensional version, the NDI-8 replaces the original [58].

The reliability and responsiveness are lower in the QuickDASH-9 compared to the DASH. This is anticipated and consistent with previous QuickDASH findings [20,21,26,32] as the reduction in items from 30 to nine is substantial.

The QuickDASH-9 mean percentage scores were found to be higher than those of the DASH. This supports previous findings that a shortened tool with improved internal consistency will show greater scale width, particularly for higher impairment conditions [21]. The choice of eleven items for the QuickDASH is based on the *a-priori *assumption drawn from the 'Spearman-Brown prophesy'. Specifically, that a minimum of eleven items is required to produce an internal consistency within the clinically accepted range of 0.90 to 0.95 [20]. This study has shown that in a shortened 9-item version, the internal consistency can remain within this range and provide a valid instrument with significant gains in practicality. However, a computational scoring aid is still required.

In both stages of this study the QuickDASH-9 showed inferior psychometric properties to the DASH and ULFI, particularly for reliability and error scores. In relation to the DASH this is outweighed by the gains in practicality and internal consistency, but not in comparison to the ULFI. These findings are reflected in the summary scores of the 'Measurement of Outcome Measures' and the 'Bot scale' that supports the preference of the QuickDASH-9 over the DASH. However, both tools remained notably lower than the ULFI on both scales which scores as the preferred instrument for both clinical and research purposes due to its practicality and lower missing responses.

### Limitations

The study investigated only outpatients presenting to primary care physical therapy practices and further research is required to clarify these findings in an inpatient setting. The findings are general and extrapolation to specific conditions must be made with caution till such conditions are individually investigated. There was a consistent difference in the QuickDASH-9 order of factor loading between the calibration and validation stages. This is most likely from differences in the samples due to the diverse range of diagnoses and duration times used in each stage.

### Strengths

The findings have broad implications for use in the general population as they are not specific to one condition or population group as participants were from general outpatient populations. Two independent population samples are used for data extraction to examine the QuickDASH-9 characteristics. The use of a consistent reference criterion, the ULFI, supports the similarity of findings in the two samples.

### Implications for Practice

The QuickDASH-9 as a valid shortened form of the DASH provides a practical approach to measurement of the upper limb. This enhanced practicality reduces the burden to both the patient and clinician, optimizing clinical practice without compromising the accuracy and error measurement capacity of the instrument.

### Implications for Research

A prospective validation of the QuickDASH-9 is required in an independent sample using an established criterion, such as the ULFI. Further investigation of the psychometric properties in samples of specific populations and conditions is also required. This could initially be investigative through extraction of responses from existing DASH and QuickDASH studies, with prospective investigation to follow. However, with the summary performance of all forms of the DASH concept shown to be lower than the ULFI, the adoption of the ULFI as a single preferred standard may be preferable.

## Conclusions

The unidimensional structure found in the proposed QuickDASH-9 is valid and consistent with the full-length DASH. This achieves the original aim of the QuickDASH, to be a shortened version with comparable or preferable psychometric properties, no item redundancy and higher practicality. The QuickDASH, with a bidimensional structure, is invalid for the production of a summated score. This shortcoming is overcome by the QuickDASH-9. Furthermore, the QuickDASH-9 eliminates item redundancy found in the DASH, improves internal consistency, completion and scoring times and enhances practicality. The QuickDASH-9 offers a viable future option for the DASH concept.

## Abbreviations

DASH: Disabilities of Arm; Shoulder and Hand questionnaire; ES: Effect size; ICC: Intraclass correlation coefficient; KS: Kolmogorov-Smirnov test for normality; n: number of participants; n^R^: number of responses; MDC: Minimal detectable change; MLE: Maximum likelihood extraction; NDI: Neck Disability Index; NRS: Numeric Rating Scale; PRO: Patient Reported Outcome; QuickDASH: Shortened 11-item version of Disabilities of Arm, Shoulder and Hand questionnaire; QuickDASH-9: Shortened 9-item version of Disabilities of Arm, Shoulder and Hand questionnaire; SD: Standard deviation; SRM: Standard response mean; ULFI: Upper Limb Functional Index.

## Competing interests

The authors declare that they have no competing interests.

## Authors' contributions

CPG is the principal investigator. He designed the study and is responsible for the protocol. CPG is also responsible for data acquisition and analysis. Together with CPG, BB and MY developed the key ideas underlying this study, interpreted the data, wrote and revised the manuscript. MM has been involved in interpretation of the data and revising the manuscript critically for shortcomings of the original QuickDASH and for the validation of the QuickDASH-9. All authors read and approved the final manuscript.

## Pre-publication history

The pre-publication history for this paper can be accessed here:

http://www.biomedcentral.com/1471-2474/10/161/prepub

## References

[B1] Angst F Goldhahn J Drerup S Aeschlimann A Schwyzer H R S Responsiveness of six outcome assessment instruments in total shoulder arthroplasty Arthritis Care Res 2008 59 3 391 398 10.1002/art.23318 18311752

[B2] Doward LC McKenna SP Defining Patient-Reported Outcomes Value Health 2004 7 S1 S4 S8 10.1111/j.1524-4733.2004.7s102.x 15367236

[B3] Cleland JA Childs JD Whitman JM Psychometric properties of the Neck Disability Index and Numeric Pain Rating Scale in patients with mechanical neck pain Arch Phys Med Rehabil 2008 89 1 69 74 10.1016/j.apmr.2007.08.126 18164333

[B4] Beaton DE Boers M Wells GA Many faces of the minimal clinically important difference (MCID): a literature review and directions for future research Curr Opin Rheumat 2002 14 2 109 114 10.1097/00002281-200203000-00006 11845014

[B5] Policy on outcome measures and treatment justification http://www.tac.vic.gov.au/upload/TAC+Physio+Chart.pdf

[B6] Management of Soft Tissue Injuries http://www.workcover.nsw.gov.au/Documents/Publications/InjuryManagementRTW/RehabilitationProviders/overview_improving_outcomes_5364.pdf

[B7] Website for the Canadian Institute for Work Health http://www.iwh.on.ca

[B8] The Clinical Framework for the Delivery of Health Services http://www.tac.vic.gov.au/upload/clinical-framework-single.pdf

[B9] Beaton DE Katz NK Fossel AH Wright JG Tarasuk V Bombardier C Measuring the whole of the parts? Validity, reliability, and responsiveness of Disabilities of the Arm Shoulder and Hand outcome measure in different regions of the upper limb J Hand Ther 2001 14 128 146 11382253

[B10] Michener LA Leggins BG A review of self-report scales for the assessment of functional limitation and disability of the shoulder J Hand Ther 2001 14 68 76 11382257 10.1016/s0894-1130(01)80036-3

[B11] Gabel CP Michener L Burkett B Neller A The Upper Limb Functional Index (ULFI): Development and Determination of Reliability, Validity and Responsiveness J Hand Ther 2006 19 3 328 349 10.1197/j.jht.2006.04.001 16861132

[B12] Hudak PL Amadio PC Bombardier C (UECG) TUECG Development of an upper extremity outcome measure: The DASH (Disabilities of the Arm, Shoulder, and Head) Am J Ind Med 1996 29 602 608 10.1002/(SICI)1097-0274(199606)29:6<602::AID-AJIM4>3.0.CO;2-L 8773720

[B13] Davis AM Beaton DE Hudak P Amadio P Bombardier C Cole C Hawker G Katz JN Makela M Marx RG Measuring disability of the upper extremity: a rationale supporting the use of a regional outcome measure J Hand Ther 1999 12 269 274 10622192 10.1016/s0894-1130(99)80063-5

[B14] MacDermid JC Tottenham V Responsiveness of the disability of the arm, shoulder, and hand (DASH) and patient-rated wrist/hand evaluation (PRWHE) in evaluating change after hand therapy J Hand Ther 2004 17 1 18 23 10.1197/j.jht.2003.10.003 14770134

[B15] Bialocerkowski A Disabilities of the arms, shoulder and hand questionnaire Aust J Physiother: Clinimetrics 2007 53 2 135 10.1016/s0004-9514(07)70050-8 17535153

[B16] Bot SD Terwee CB Windt DA van der Bouter LM Dekker J de Vet HC Clinimetric evaluation of shoulder disability questionnaires: a systematic review of the literature Ann Rheum Dis 2004 63 4 335 341 10.1136/ard.2003.007724 15020324 PMC1754942

[B17] Mousavi SJ Parnianpour M Abedi M Askary-Ashtiani A Karimi A Khorsandi A Mehdian H Cultural adaptation and validation of the Persian version of the Disabilities of the Arm, Shoulder and Hand (DASH) outcome measure Clin Rehabil 2008 22 8 749 757 10.1177/0269215508085821 18678575

[B18] Gummesson C Atroshi I Ekdahl C The disabilities of the arm, shoulder and hand (DASH) outcome questionnaire: longitudinal construct validity and measuring self-rated health change after surgery BMC Musculoskelet Disord 2003 4 11 10.1186/1471-2474-4-11 PMC165599 12809562

[B19] Development and testing of the DASH and Quick-DASH Outcome Measure Instruments and the DASH User's Manual http://www.dash.iwh.on.ca

[B20] Beaton DE Wright JG Katz JN Group UEC Development of the QuickDASH: comparison of three item-reduction approaches J Bone Joint Surg Am 2005 87 5 1038 1046 10.2106/JBJS.D.02060 15866967

[B21] Gummesson C Ward MM Atroshi I The shortened disabilities of the arm, shoulder and hand questionnaire (QuickDASH): validity and reliability based on responses within the full-length DASH BMC Musculoskelet Disord 2006 7 4 10.1186/1471-2474-7-44 PMC1513569 16709254

[B22] Wyatt MC Veale GA Early return to work following open carpal tunnel decompression in lamb freezing workers J Hand Surg Eur Vol 2008 33 4 440 444 10.1177/1753193408090145 18687831

[B23] Atroshi I Lyrén PE Gummesson C The 6-item CTS symptoms scale: a brief outcomes measure for carpal tunnel syndrome Qual Life Res 2009 18 3 347 358 10.1007/s11136-009-9449-3 19229657

[B24] Mintken PE Glynn P Cleland JA Psychometric properties of the shortened disabilities of the Arm, Shoulder, and Hand Questionnaire (QuickDASH) and Numeric Pain Rating Scale in patients with shoulder pain J Shoulder Elbow Surg 2009 18 6 920 6 Epub 2009 Mar 17 10.1016/j.jse.2008.12.015 19297202

[B25] Imaeda T Toh S Wada T Uchiyama S Okinaga S Kusunose K Sawaizumi T Impairment Evaluation Committee, Japanese Society for Surgery of the Hand Validation of the Japanese Society for Surgery of the Hand Version of the Quick Disability of the Arm, Shoulder, and Hand (QuickDASH-JSSH) questionnaire J Orthop Sci 2006 11 3 248 253 10.1007/s00776-006-1013-1 16721524 PMC2778693

[B26] Fayad F Lefevre-Colau MM Gautheron V Macé Y Fermanian J Mayoux-Benhamou A Roren A Rannou F Roby-Brami A Revel M Reliability, validity and responsiveness of the French version of the questionnaire Quick Disability of the Arm, Shoulder and Hand in shoulder disorders Man Ther 2009 14 6 206 12 Epub 2008, Apr 23 10.1016/j.math.2008.01.013 18436467

[B27] Meads D Doward L McKenna S Fisk J Twiss J Eckert B The development and validation of the Unidimensional Fatigue Impact Scale (U-FIS) Mult Scler 2009 in press 10.1177/1352458509106714 19556314

[B28] Fabrigar LR Wegener DT MacCallum RC Strahan EJ Evaluating the use of exploratory factor analysis in psychological research Psychol Methods 1999 4 272-299

[B29] Matheson LN Melhorn JM Mayer TG Theodore BR Gatchel RJ Reliability of a visual analog version of the QuickDASH J Bone Joint Surg Am 2006 88 8 1782 1787 10.2106/JBJS.F.00406 16882902

[B30] Wu A Edgar DW Wood FM The QuickDASH is an appropriate tool for measuring the quality of recovery after upper limb burn injury Burns 2007 33 7 843 849 10.1016/j.burns.2007.03.015 17686586

[B31] Stover B Silverstein B Wickizer T Martin DP Kaufman J Accuracy of a disability instrument to identify workers likely to develop upper extremity musculoskeletal disorders J Occ Rehab 2007 17 2 227 245 10.1007/s10926-007-9083-2 17487573

[B32] Abramo A Kopylov P Tagil M Evaluation of a treatment protocol in distal radius fractures: a prospective study in 581 patients using DASH as outcome Acta Orthop 2008 79 3 376 385 10.1080/17453670710015283 18622842

[B33] Gabel CP Michener LA Melloh M Burkett BB The Upper Limb Functional Index (ULFI) - Validation with Improved Psychometric and Practical Characteristics Using a Three-Point Likert Scale J Hand Ther 2010 http://www.jhandtherapy.org/article/S0894-1130(09)00127-6/abstract 10.1016/j.jht.2009.09.007 19963344

[B34] Gabel CP Burkett B Yelland M Short Version Outcome Measures: Balancing Fidelity and Practicality Phys Ther Rev 2009 14 4 221 225 10.1179/174328809X452890

[B35] McConnell S Beaton DE Bombardier C The DASH Outcome Measure User's Manual 1999 Toronto, Canada: Institute for Work and Health

[B36] Gabel P Barden L Burkett B Neller L Integrating Injury Screening with Measurement and Monitoring: A Conceptual Approach Using A Patient Global Assessment of Body and Limbs Scale South African Journal of Physiotherapy 2006 62 4 2 8

[B37] O'Halloran J Miller GC Britt H Defining chronic conditions for primary care with ICPC-2 Fam Pract 2004 21 4 381 386 10.1093/fampra/cmh407 15249526

[B38] Shrout PE Fleiss JL Intraclass correlations: uses in assessing rater reliability Psychol Bull 1979 86 2 420 428 10.1037/0033-2909.86.2.420 18839484

[B39] Liang MH Evaluating measurement responsiveness J Rheumatol 1995 22 1191 1192 7674254

[B40] Liang MH Longitudinal construct validity: establishment of clinical meaning in patient evaluative instruments Med Care 2000 38 9 Suppl II84 90 10982093

[B41] Jacobson NS Roberts LJ Berns SB McGlinchey JB Methods for defining and determining the clinical significance of treatment effects: description, application and alternatives J Consult Clin Psychol 1999 67 300 307 10.1037/0022-006X.67.3.300 10369050

[B42] Cronbach LJ Coefficient alpha and the internal structure of tests Psychometrika 1951 16 297 334 10.1007/BF02310555

[B43] Nunnally JC Bernstein IH Psychometric Theory 1994 New York: McGraw-Hill

[B44] Fields A Discovering Statistics using SPSS 2005 2 London: SAGE Publications Ltd

[B45] Stevens JP Applied multivariate statistics for the Social Sciences 1992 2 Hillsdale, NJ: Erlbaum

[B46] Cattell RB The scree test for the number of factors Multivariate Behavioural Research 1966 1 245 276 10.1207/s15327906mbr0102_10 26828106

[B47] Kaiser HF The application of electronic computers to factor analysis Educ Psychol Meas 1960 20 141 151 10.1177/001316446002000116

[B48] Dawson B Trapp R Basic and Clinical Biostatistics 2001 2 Sydney: Mcgraw Hill

[B49] Doak CC Doak LG Root JH Teaching patients with low literacy skills 1996 2 Philadelphia: J.B. Lippincott

[B50] Paasche-Orlow MK Taylor HA Brancati FL Readability Standards for Informed-Consent Forms as Compared with Actual Readability N Engl J Med 2003 348 8 721 726 10.1056/NEJMsa021212 12594317

[B51] De Vet HC Ostelo RW Terwee CB Roer N van der Knol DL Beckerman H Boers M Bouter LM Minimally important change determined by a visual method integrating an anchor-based and a distribution-based approach Qual Life Res 2007 16 1 131 142 10.1007/s11136-006-9109-9 17033901 PMC2778628

[B52] Lee EW Chung MM Li AP Lo SK Construct validity of the Chinese version of the disabilities of the arm, shoulder and hand questionnaire (DASH-HKPWH) J Hand Surg [Br] 2005 30 1 29 34 15620488 10.1016/j.jhsb.2004.09.010

[B53] MacDermid JC Wessel J Humphrey R Ross D Roth JH Validity of self-report measures of pain and disability for persons who have undergone arthroplasty for osteoarthritis of the carpometacarpal joint of the hand Osteoarthritis Cartilage 2007 15 5 524 530 10.1016/j.joca.2006.10.018 17161960

[B54] Themistocleous GS Goudelis G Kyrou I Chloros GD Krokos A Galanos A Gerostathopoulos NE Soucacos PN Translation into Greek, cross-cultural adaptation and validation of the Disabilities of the Arm, Shoulder, and Hand Questionnaire (DASH) J Hand Ther 2006 19 3 350 357 10.1197/j.jht.2006.04.014 16861133

[B55] Reeve B Hays RD Bjorner JB Cook KF Crane PK Teresi JA Thissen D Revicki D Weiss DJ Hambleton RK Psychometric Evaluation and Calibration of Health-Related Quality of Life Item Banks: Plans for the Patient-Reported Outcomes Measurement Information System (PROMIS) Med Care 2007 45 5 (Suppl 1) S22 S31 The Patient-Reported Outcomes Measurement Information System (PROMIS) Overview and Developmental Work) 10.1097/01.mlr.0000250483.85507.04 17443115

[B56] Coste J Guillemin F Pouchot J Fermanian J Methodological approaches to shortening composite measurement scales J Clin Epidemiol 1997 50 3 247 252 10.1016/S0895-4356(96)00363-0 9120523

[B57] Young SB Aprill C Braswell J Ogard W Richards JS McCarthy JP Psychological Factors and Domains of Neck Pain Disability Pain Med 2009 10 2 310 318 10.1111/j.1526-4637.2009.00571.x 19284486

[B58] Velde G van der Beaton D Hogg-Johnston S Hurwitz E Tennant A Rasch analysis provides new insights into the measurement properties of the neck disability index Arthritis Rheum 2009 61 4 544 551 10.1002/art.24399 19333989

